# When School and Family Convey Different Cultural Messages: The Experience of Turkish Minority Group Members in France

**DOI:** 10.5334/pb.283

**Published:** 2016-05-25

**Authors:** Cristina Aelenei, Céline Darnon, Delphine Martinot

**Affiliations:** 1 Université Clermont Auvergne, Université Blaise Pascal, FR; 2 CNRS, UMR 6024, LAPSCO, FR

**Keywords:** School-culture, Turkish-minority, values, individualism, collectivism

## Abstract

The present studies aim to compare the cultural values promoted by the French educational system and the Turkish families living in France to their youngsters. Because of their collectivist background Turkish immigrants may convey less individualistic values to their children compared to French parents and teachers. However, Turkish students may become more individualistic as they are socialized in the school system. In study 1 (N = 119), French school teachers, French parents, and Turkish-origin parents had to resolve six dilemmas by choosing either an individualistic or a collectivistic response-option. As expected, French teachers emphasized individualism more than Turkish parents, but not more than French parents. In Study 2 (N = 159), similar dilemmas were presented to French and Turkish-origin pupils. In elementary school, Turkish children were less individualistic than French-born children, but this gap was reduced in high school.

The dynamic relationship between school and family has become a subject of major interest in recent decades due to the increasing cultural diversity of the student body in Western school systems. With the proliferation of studies on this topic, a cultural discontinuity hypothesis between the values promoted in school versus those at home, particularly for minority students, has emerged. Such a discontinuity may be linked to various academic challenges (for a review, see Tyler et al., 2008). Recent studies have refined the cultural discontinuity literature (e.g., Baysu, Phalet, & Brown, 2014; Shook & Fazio, 2008) by suggesting that an adaptive exposure to the school’s predominant values is beneficial to minority children. Indeed, Shook and Fazio (2008) found that the African American students randomly assigned to White roommates obtained better GPAs than those assigned to same-race roommates. In the same vein, Baysu, Phalet, and Brown (2014) demonstrated that the number of cross-group friendships was positively related to Turkish students’ school attainment in Belgium and Austria, leading the authors to conclude that: “*Majority group friends may facilitate access to culturally grounded knowledge and behavioural repertoires which are typically valued in the school context and generally lacking in immigrant families”* (p. 342). Indeed, as formally argued by Bourdieu, Passeron, and Nice (1990) and more recently by Tyler et al. (2008), the school culture is not neutral; its cultural values are more or less in line with the values promoted within families. Following the republican principle of universalism, the French school institution plays down the cultural minority status in students’ school experiences (Oberti, 2008). Yet, while proclaiming this policy, the French school institution may in fact create a cultural environment that can be quite different from the home culture of minority families.

The purpose of this research is twofold. First, it aims to shed light on the cultural values promoted by the French school system and examine the extent to which these values match the cultural values promoted by two types of families, the French families, and the Turkish families living in France (i.e., 1.2% of the population; INSEE, 2012). Second, it examines the extent to which the children themselves endorse these values as a function of their origin (French or Turkish) and their experience of the French school system.

Turkish culture appears to be a collectivistic culture, emphasizing groups’ interests over those of separate individuals (Hofstede, 1983; Triandis, Chen, & Chan, 1998). In the index created by Diener, Gohm, Suh, and Oishi (2000), Turkey ranked as the fourth most collectivistic country after China, South Korea, and Nigeria, whereas France was considered as one of the most individualistic countries (11^th^ out of 43). On Hofstede’s index of individualism, Turkey ranked 37^th^ out of 50, whereas France ranked 11^th^. Moreover, according to the immigrant interdependence hypothesis (Ayçiçegi-Dinn & Caldwell-Harris, 2011), in a migration context, individuals may become more collectivistic because they need to rely on the small community to which they belong in order to overcome the difficulties inherent in settling down in a new country. Consequently, the attachment to collectivistic values might be particularly important for Turkish families living in France. On the other hand, the French school institution should reflect the mainstream culture (Bourdieu et al., 1990), where individualism is a social norm.

Thus, in Study 1, we examine whether Turkish parents—by conserving their collectivistic values inherited from their home culture and accentuated in the immigration context—differ from the school values more than French families do. In Study 2, we examine which values Turkish-origin children endorse compared to their French-born counterparts when immersion in the school context is still relatively new (i.e., elementary school) as well as after several years of schooling (i.e., junior high school).

## Study 1

### Method

#### Participants

The study was conducted in two schools of a small French city, selected for their high population of Turkish-origin pupils (e.g., 60% of children in the elementary school). Nineteen elementary and junior high school teachers (3 in elementary school, 16 in junior high school; 15 women, 4 men; *M*_age_ = 34.68) and 100 parents—23 Turkish (11 women, 12 men; *M*_age_ = 41.16) and 77 French (55 women, 22 men; *M*_age_ = 41.50)—participated. The two school directors and a literature professor distributed and collected the questionnaires to all school professors and to parents via their children.

#### Procedure

Following Triandis, Chen, and Chan (1998), we created a measure consisting of six dilemmas that required participants to choose between two proposals: one describing an individualistic attitude and another describing a collectivistic one. In constructing our measure, we used items from a series of validated scales measuring individualism and collectivism (e.g., Singelis, Triandis, Bhawuk, & Gelfand, 1995), which we exemplified either by a specific scenario or by translating them into a school-related context.

We followed Triandis et al.’s (1998) methodological guidelines to verify the validity of our measure. First, we assessed the conceptual validity of our items by having five graduate student judges indicate which responses to each scenario represented individualistic or collectivistic judgments, after being provided with the definitions of individualism and collectivism. All propositions showed a complete agreement across judges. Second, given that the standardized Cronbach’s coefficient α tends to critically underestimate the true reliability of a measure containing a small number of heterogeneous items—more so when the items are dichotomous (Sun et al., 2007)—we assessed the reliability of our measure by reporting the correlation between the computed scores of the two halves of our measure. More specifically, two scores were obtained for each participant by dividing the questionnaire into equivalent halves (the odd items versus the even items). This correlation was *r* = 0.31 (*p* = .023). The dilemmas were adapted for teachers and parents, who were asked to indicate which of the two response options they would communicate as desirable to their pupils/children (e.g., “If I think of my students/children, I encourage them to succeed in life, because: a. They will be able in this way to make their own life and do what they like. b. They could help their family(ies), and it would be good for the entire society.”).

Individualistic response choices were coded +1 whereas collectivistic answers were coded 0. The mean score could range from 0 (strong collectivistic preference) to 1 (strong individualistic preference), *M* = 0.77 and *SD* = 0.18.

### Results and Discussion

Because of a highly skewed distribution and unequal sample sizes, a Kruskal-Wallis H test was performed for the omnibus effect, followed by Mann-Whitney tests for pairwise comparisons, with a Bonferroni correction. The Kruskal-Wallis H test was significant: *H*(2) = 12.22, *p* < .01, η² = .10. Turkish parents (*M* = 0.66; *SD* = 0.22) scored lower on individualism than teachers (*M* = 0.86; *SD* = 0.08) and French parents (*M* = 0.78; *SD* = 0.17). Teachers differed significantly from Turkish parents, *Z*(40) = 3.32, *p* < .01, and French parents differed significantly from Turkish parents, *Z*(98) = 2.46, *p* < .05, but not from teachers, *Z*(94) = –2.00, *p* > .10, *ns*. Building on these findings, Study 2 investigated the expression of the I/C among Turkish-origin versus French-born pupils and examined if this expression was a function of the school level (primary versus secondary).

## Study 2

According to the immigrant interdependence hypothesis (Ayçiçegi-Dinn & Caldwell-Harris, 2011), Turkish-origin children might preserve their family values, which are less individualistic than the values of French-born families (Study 1). Thus, we suggest that Turkish-origin pupils may express less individualistic attitudes than their French-born classmates, regardless of their school level (H1). However, as demonstrated by recent studies (Baysu et al. 2014, Shook & Fazio, 2008), because of an adaptive exposure to the school’s predominant values, these pupils might acquire individualistic values they are gradually provided within school. Therefore, an alternative hypothesis proposes that the difference between French and Turkish-origin students in the endorsement of individualistic values should be higher among young students (e.g., elementary students) than among older students (e.g., junior high school students) (H2).

### Method

#### Participants

Our sample counted 159 elementary (*N* = 51) and junior high school (*N* = 108) students, (82 girls; 77 boys) recruited from the same schools as in Study 1. Fifty-eight were identified as Turkish (32 in elementary school; 26 in junior high school) and 101 as French (19 in elementary school; 82 in junior high school). All students at the primary school participated. At the secondary school, we had access to a panel of randomly chosen classes, covering all grades (5^th^ to 9^th^). The stop rule in data collection was to have at least 100 participants.

#### Procedure

We used the same social dilemmas as in Study 1, but adapted them to students. We assessed the reliability of our measure using the same procedure as in Study 1, obtaining a correlation *r* = 0.44 (*p* < .001). The students’ mean score could range from 0 (strong collectivist preference) to 1 (strong individualistic preference), *M* = 0.55, *SD* = 0.24.

### Results and Discussion

**Figure 1:**
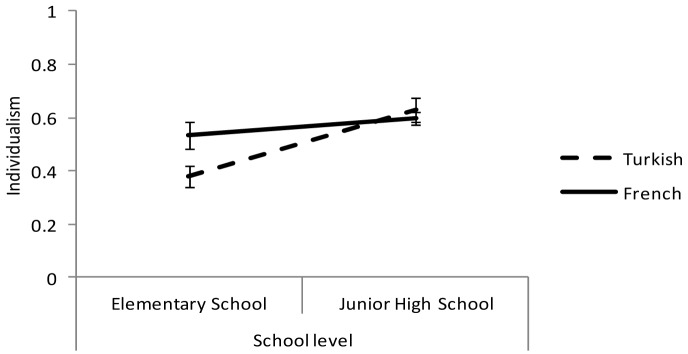
Degree of individualism as a function of school level and students’ origin. Error bars represent standard errors.

Regression analyses were conducted to analyze the data. In the preliminary analyses, gender, as well as its interactions with the independent variables (i.e., school level and origin,) was entered into the analyses. Because the inclusion of gender was found to neither produce significant effects nor alter the effects of our independent variables, it was dropped from the final analyses (Yzerbyt, Muller, & Judd, 2004). Thus, the regression model contained three predictors: students’ origin (–1 for Turkish, 1 for French), school level (–1 for elementary school, 1 for junior high school), and the interaction between these two variables. Regressing individualism endorsement on the predictors did not support H1. Indeed, the main effect of students’ origin was not significant: *t*(155) = 1.49, *p* = .138, *ns*. The main effect of school level was significant, indicating that junior high school students (*M* = 0.60, *SD* = 0.23) endorsed individualism to a greater extent than elementary school students (*M* = 0.43, *SD* = 0.22): *b* = 0.08, *SE* = 0.02, *t*(155) = 3.73, *p* < .001, η²_p_ = .08. However, the effect of school level was moderated by students’ origin. Indeed, in line with H2, the interaction between school level and students’ origin indicated that the difference between French and Turkish pupils decreased from the elementary to secondary school level: *b* = –0.05, *SE* = 0.02, *t*(155) = 2.20, *p* = .029, η²_p_ = .03. It was significant in elementary school, *b* = 0.08, *SE* = 0.03, *t*(155) = 2.34, *p* = .020, η²_p_ = .03, but not in junior high school, *b* = –0.02, *SE* = 0.03, *t* < 1, *p* = .561, *ns* (Figure 1). Furthermore, Turkish pupils were significantly less individualistic in elementary school than in junior high school, *b* = 0.12, *SE* = 0.03, *t*(155) = 4.13, *p* < .001, η²_p_ = .10, whereas this difference was not significant for French pupils, *b* = 0.03, *SE* = 0.03, *t*(155) = 1.09, *p* = .275, *ns*.

These findings suggest that, upon entering elementary school, Turkish children are steeped in the cultural marks displayed in their family, thereby expressing less individualism than their French counterparts. Moreover, because Turkish immigrants in France are organized in tightly knit communities (Demirel, 2008), even outside their nuclear family, Turkish-origin children are likely to be predominantly exposed to collectivistic values. However, once they integrate into the French school context, they are exposed to the highly individualistic values promoted by teachers (Study 1). Hence, a process of cultural learning may explain why the cultural difference between Turkish and French students observed at the primary school level is attenuated at the secondary school level.

## General Discussion

These two studies document how Turkish-origin students experience the school system in France in terms of cultural values. Study 1 showed the discrepancy between Turkish families’ values and French school values. These results are particularly important because the teacher sample came from two schools with high percentages of Turkish-origin pupils, hence, working directly within a culturally diverse student body. These results also show that Turkish immigrants in France carry on values from their collectivistic cultural heritage and are willing to pass them on to their children. Moreover, at their schooling age, these children are integrated into the French school system, which exposes them to the individualistic values promoted by teachers. Examining the consequences of this cultural exposure, Study 2 showed that Turkish-origin students expressed less individualistic attitudes than their French classmates in elementary school. This difference was reduced in junior high school, where Turkish-origin pupils were as individualistic as their French-origin classmates. On the basis of Berry’s (1997) work, two acculturation strategies are conceivable to explain such a result. First, Turkish-origin children may adopt a general individualistic approach in all their life spheres, not only in the school context. This defines an acculturation strategy of broad assimilation into the dominant values of the host society. However, another acculturation strategy (i.e., integration) implies an interest in both maintaining a sense of one’s original culture while adapting to the dominant values in the host society, particularly in the officially institutionalized contexts (e.g., school, job). Thus, while endorsing the individualistic values in the school context, Turkish-origin children may display a collectivistic cultural maintenance in more private spheres (e.g., the extended family, the ethnic community).

Some limitations merit discussion. First, because any question perceived as being related to ethnicity in the French context generates quite a large amount of refusals from individuals, the sample sizes for teachers and parents were not as large as expected. Moreover, one may wonder whether the differences observed in the two studies are due exclusively to culture or also to religious values. Replicating the present findings while integrating measures of religious values and of hypothesized processes (for instance, assimilation or integration strategy of acculturation) would nicely complement the present findings. Moreover, the processes explaining how Turkish pupils became more individualistic throughout their school years should be examined, specifically by using a longitudinal design.

Finally, the present results support the idea that the French school context can contribute to a more successful integration of Turkish children into French society by teaching them the dominant values. However, Turkish-origin children arrive at elementary school with a less individualistic approach than French-born children. Such different cultural backgrounds are likely to produce certain difficulties in understanding the norm. For instance, Triandis and Suh (2002) found that, in collectivistic cultures, asking the teacher a question is against the norm. Therefore, it seems important to keep French elementary school teachers informed of these cultural differences.

## Authors’ note

Cristina Aelenei, Céline Darnon, & Delphine Martinot, Université Clermont Auvergne, Université Blaise Pascal, Laboratoire de Psychologie Sociale et Cognitive, BP 10448, F-63000 CLERMONT-FERRAND, FRANCE. Part of this work was supported by the Swiss National Foundation (Sinergia CRSII1 141872).
